# Effect of Blood Exposure on Push-Out Bond Strength of Four Calcium Silicate Based Cements 

**DOI:** 10.22037/iej.2017.38

**Published:** 2017

**Authors:** Kazem Ashofteh Yazdi, Behnam Bolhari, Tohid Sabetmoghaddam, Naghmeh Meraji, Mohammad Javad Kharazifard

**Affiliations:** a *Department of Endodontics, School of Dentistry, Tehran University of Medical Sciences, Tehran, Iran; *; b * Private Practice, Tehran, Iran; *; c * Dental Research Center, School of Dentistry, Tehran University of Medical Sciences, Tehran, Iran*

**Keywords:** Biodentine, Blood, Calcium-Enriched Mixture Cement, Endosequence Root Repair Material, Mineral Trioxide Aggregate

## Abstract

**Introduction:**

The purpose of this study was to compare the push-out bond strength of white ProRoot Mineral Trioxide Aggregate (MTA), Biodentine, calcium-enriched mixture (CEM) cement and Endosequence Root Repair Material (ERRM) putty after exposure to blood.

**Methods and Materials:**

A total of 96 root dentin slices with a standardized thickness of 1.00±0.05 mm and standardized canal spaces were randomly divided into 4 main experimental groups (*n*=24) according to the calcium silicate based cement (CSC) used: white ProRoot MTA, CEM Cement, ERRM Putty and Biodentine. Specimens were exposed to whole fresh human blood and then subdivided into two subgroups depending on the exposure time (24 or 72 h). Push-out bond strength was measured using a universal testing machine. Failure modes were examined under a light microscope under ×10 magnification. Data were analyzed using the two-way ANOVA test.

**Results:**

Biodentine exhibited the highest values regardless of the exposure time. The lowest push-out strength values were seen in white ProRoot MTA and CEM cement in both post exposure times. After exposure to blood, the push-out bond strength of all materials increased over time. This increase was only statistically significant in white ProRoot MTA and ERRM specimens. The dominant failure mode in all CSCs was the adhesive mode.

**Conclusion:**

Biodentine showed the highest values of push-out bond strength and may be better options for situations encountering higher dislocation forces in a short time after cement application.

## Introduction

To date numerous calcium silicate based cements (CSCs) have been introduced as alternatives for mineral trioxide aggregate (MTA) with the aim of overcoming the disadvantages of this cement such as difficult handling properties and tooth discoloration [1-5]. For instance Biodentine was introduced as a fast setting CSC with high compressive strength [6, 7], Endosequence Root Repair Material (ERRM) and calcium-enriched mixture (CEM) cement were introduced as white CSCs with no discoloration potential and better handling characteristics [8, 9]. The same clinical applications as MTA have been suggested for these CSCs [10, 11]. During their clinical applications, CSCs are in contact with blood and body fluids. Several studies have shown that exposure to blood negatively affects some physical properties of MTA and other CSCs [12-15].

CSCs can also be exposed to dislocating forces such as condensational forces during restoration placement or masticatory forces when used as root-end filling. Resistance to these dislocating forces is a required characteristic for CSCs [16]. Moreover, they are in contact with body fluids especially blood [17]. The purpose of this study was to compare the push-out bond strength of four CSCs, including white ProRoot MTA, Biodentine, CEM cement and ERRM putty after exposure to blood. The null hypothesis was that the push-out bond strength of four evaluated CSCs would not differ.

## Materials and Methods

Whole fresh human blood was obtained by phlebotomy using a 23-gauge needle from a healthy volunteer member of the research group who gave informed consent in accordance with the declaration of Helsinki ethical principles [18].

***Specimen preparation:***

A total of 96 single-rooted human mandibular premolars extracted due to periodontal problems stored in 0.5% Chloramine T [19] were selected. Teeth with severe curvature, caries, cracks, and resorptive defects in the roots were excluded. After mounting the teeth in acrylic resin, the middle third of the roots were sectioned perpendicular to the long axis into a 1.00±0.05 mm thick slice using a water cooled diamond blade on a cutting machine (Mecatome, Presi, France). To ensure having the proper thickness, each slice was measured using a digital caliper (Digimatic, Mitutoyo Corporation, Tokyo, Japan).

The lumen of the slices was instrumented using Gates-Glidden drills (Dentsply Maillefer, Ballaigues, Switzerland), sizes 2-5, to obtain a standardized diameter of 1.3 mm. The specimens were immersed in 17% EDTA (Meta Biomed Co., Ltd., Mandaluyong, Korea) for 3 min, followed by immersion in 1% sodium hypochlorite for the same duration for smear layer removal. Afterwards they were washed in distilled water and dried. Obtained specimens were randomly divided into four groups (*n*=24) according to the CSC applied: White ProRoot MTA (Dentsply Tulsa Dental, Johnson city, TN, USA), CEM Cement (BioniqueDent, Tehran, Iran), ERRM Putty (Brasseler USA, Savannah, GA, USA) and Biodentine (Septodont, Saint Maur des Fosses, Cedex, France).

In order to standardize the sample preparation of white ProRoot MTA and CEM Cement, 1 g of each powder and 0.33 g distilled water were placed in plastic mixing capsules [20]. Biodentine is provided as a powder containing capsule and single dose liquid container. According to the manufacturer’s instructions five drops of the liquid in the container was added to the capsule containing powder. 

All encapsulated materials were then mechanically mixed for 30 sec at 4500 rpm [21] using an amalgamator (Silamat; Ivoclar Vivadent AG, Liechtenstein). ERRM putty is premixed by the manufacturer. Tooth slices were placed on a glass slab. After preparation, all materials were then placed into the canal space, adapted gently into the standardized root canal lumens. Afterwards, a blood-soaked gauze was placed beneath the specimens and another gauze soaked in distilled water was placed above them. Specimens in each group were divided into two subgroups (*n*=12) according to the duration of exposure to blood (24 or 72 h) prior to push-out texting. All specimens were incubated at 37^o^C in fully saturated conditions. Apical and coronal aspects of each slice were then digitally photographed after incubation and before push-out testing. Afterwards, the circumferences of the filling material from the coronal and apical aspects of each slice were calculated using an AutoCAD software program (version 16.0, Autodesk, Inc., San Rafael, CA, USA). The thickness of the root slices were also measured using a digital caliper. 

***Push-out test***

After the exposure periods, specimens were submitted to the push-out test. They were placed in a 1-mm diameter cylindrical stainless steel plunger. Loading was performed on a universal testing machine (Z050, Zwick/Roell, Ulm, Germany) at a speed of 0.5 mm/min in an apical-coronal direction [22, 23]. The bond strength was determined by a computer software program connected to the universal testing machine. The maximum load applied to the filling material before debonding was recorded in Newton (N). The interfacial area (mm^2^) was calculated as follows: (coronal circumference + apical circumference)/2 × thickness [24].

Then the bond strength in megapascals (MPa) was calculated as follows: load at failure (N)/interfacial area (mm^2^). After the bond strength test was performed, both sides of the root slices were examined under a light microscope (Carl Zeiss, Oberkochen, Germany) under ×10 magnification to determine the failure mode. Modes of bond failure were considered as follows: adhesive; at filling material-dentin interface, cohesive; within filling material, and mixed failure.

The data were analyzed using the two way ANOVA and Tamhane post hoc test. The significance level was set at 0.05.

## Results

**Figure 1:**
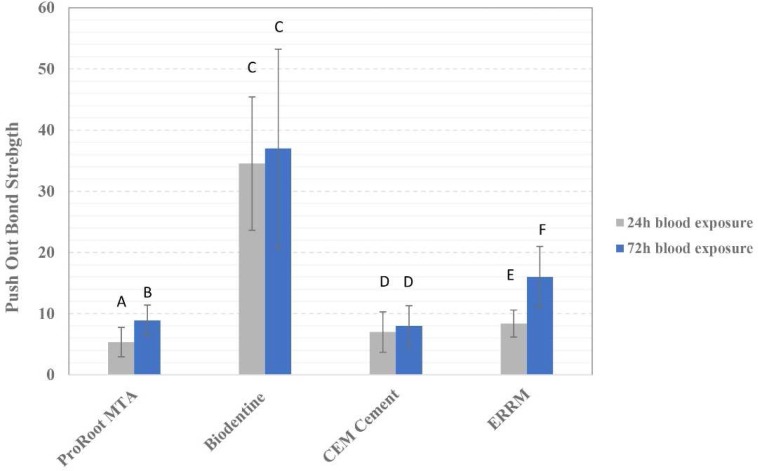
Push-out bond strength of experimental groups. Groups identified by the same superscript letters are not significantly different

The mean push-out bond strength values of each experimental group is shown in Figure 1. The highest and lowest push-out bond strength values were seen in 72-h Biodentine samples (37.03±16.16) and 24-h white ProRoot MTA (5.34±2.37) specimens, respectively.

The push-out bond strength of all CSCs exposed to blood increased over time; however, this increase was only statistically significant in white ProRoot MTA and ERRM specimens (*P*<0.05). Biodentine exhibited the highest push-out bond strength values regardless of blood exposure time. 

The percentage of adhesive, cohesive and fixed modes of fractures in each subgroup are shown in Table 1. Adhesive fracture was the predominant fracture mode seen in all experimental groups. 

## Discussion

CSCs undergo dislocating forces such as forces due to mastication and condensation of restorative materials during their clinical applications [16, 25, 26]. Resistance to these dislocating forces, especially in clinical situation such as exposure to blood and body fluids, can be an important factor for the success of endodontic treatments. Previous studies have reported the amount of condensation forces applied during amalgam placement to vary from 1.9 up to 15 MPa depending on the plugger size [17, 27]. 

In clinical conditions which CSCs are applied such as vital pulp therapy, perforation repair and root end filling, these cements inevitably encounter blood. Many studies have confirmed that blood exposure can negatively affect many properties of these CSCs [12-15] such as push-out bond strength [13, 15, 28]. Cells and proteins in blood such as albumin, can easily occlude the dentinal tubules and create gaps between the repair material and dentin walls [29]. This occlusion can adversely affect the apatite formation at the cement-dentine interface and the formation of the “mineral infiltration zone” and consequently, reduce the push-out bond strength of these CSCs. Milani *et al.* [30] reported a significant increase in marginal gaps between dentin and MTA when exposed to blood. Furthermore, blood exposure has been shown to cause air entrapment [31] and increased porosity [29] in CSCs. It is well known that blood exposure negatively affects the push-out bond strength of CSCs [13, 15, 28] and in clinical situations these cements never encounter distilled water. As a result, in the current study we compared the push-out bond strength of four CSCs only when exposed to blood. 

According to the results of the current study Biodentine exhibited the highest push-out bond strength values. The push-out bond strength of this CSC increased after 72 h but this difference was not statistically significant. This was consistent with previous studies [32]. As this cement is fast setting, it is expected to reach its maximum strength and properties earlier than other CSCs and to be less affected by environmental conditions such as blood exposure. Furthermore, smaller particle size in Biodentine may contribute to the formation of tag-like structures and better micromechanical adhesion to dentine [33]. The higher push-out bond strength values of Biodentine makes it a better option for situations encountering higher dislocation forces. A clinical example for this is vital pulp therapy, specifically in posterior teeth. Biodentine shows promising results in terms of being stable under condensation forces of the restorative materials and also chewing forces.

The lowest push-out strength values were seen in white ProRoot MTA and CEM cement in both exposure times evaluated in this study. Their push-out values increased over time but this increase was only statistically significant for white ProRoot MTA and not for CEM cement. ERRM also exhibited low push-out values but they significantly increased over time. Contrary to Biodentine, these cements exhibit extended setting time [34, 35]; therefore, can be highly influenced by environmental conditions and can reach their maximum strength and properties in a longer time interval. Considering the low push-out bond values of this cement this cement might be a better clinical choice for vital pulp therapy in anterior teeth where the chewing forces are not as great as posterior teeth and discoloration potential is a concern.

Considering the increase in the push-out bond strength of all four CSCs evaluated which was consistent with previous studies [15, 36], the negative effects of blood on the push-out bond strength may be compensated over time. 

The dominant fracture mode in all CSCs in this study was the adhesive mode. These findings were consistent with the results of some previous studies [15, 28, 37]. Adhesive mode of fracture indicates that the bonding of these CSCs to dentin is weaker than the cohesive strength of the materials themselves. This may be a result of occlusion of dentinal tubules and gap formation between the CSCs and dentinal walls consequent to blood exposure [29]. Rahimi *et al.* [13] reported the mixed mode of failure as the dominant one. Inconsistency between the results of our study with theirs may be due to differences in methodologies. This study reproduced extreme blood exposure conditions as they contaminated the internal walls of the cavity with blood whereas in our study the materials were only exposed to blood on one surface.

**Table 1:** Percentage of different modes of fractures seen in each subgroup

**Mode of fracture (%)**	**ProRoot MTA**		**Biodentine**		**CEM Cement**		**ERRM**	
	**24 h**	**72 h**	**24 h**	**72 h**	**24 h**	**72 h**	**24 h**	**72 h**
**Adhesive fracture **	66.7	75	50	66.7	50	66.7	50	58.3
**Cohesive fracture**	33.3	25	33.3	25	33.3	25	33.3	25
**Mixed fracture**	0	0	16.7	8.3	16.7	8.3	16.7	16.7

## Conclusion

Biodentine showed the highest values of push-out bond strength when exposed to blood. As exposure to blood is inevitable in clinical indications of CSCs, fast setting cements such as Biodentine may be a better option for situations encountering higher dislocation forces in a short time after cement application. The negative effects of blood exposure on CSCs seems to be reversible overtime.
